# A User-Centered Approach to an Evidence-Based Electronic Health Pain Management Intervention for People With Chronic Pain: Design and Development of EPIO

**DOI:** 10.2196/15889

**Published:** 2020-01-21

**Authors:** Ingrid Konstanse Ledel Solem, Cecilie Varsi, Hilde Eide, Olöf Birna Kristjansdottir, Elin Børøsund, Karlein M G Schreurs, Lori B Waxenberg, Karen E Weiss, Eleshia J Morrison, Mette Haaland-Øverby, Katherine Bevan, Heidi Andersen Zangi, Audun Stubhaug, Lise Solberg Nes

**Affiliations:** 1 Department of Digital Health Research Division of Medicine Oslo University Hospital Oslo Norway; 2 Institute of Clinical Medicine Faculty of Medicine University of Oslo Oslo Norway; 3 Science Centre Health and Technology University of South-Eastern Norway Drammen Norway; 4 Norwegian National Advisory Unit on Learning and Mastery in Health Oslo University Hospital Oslo Norway; 5 Centre for eHealth and Wellbeing Research University of Twente Enschede Netherlands; 6 Department of Clinical and Health Psychology University of Florida Gainesville, FL United States; 7 Department of Anesthesiology and Pain Medicine School of Medicine University of Washington Seattle, WA United States; 8 Department of Psychiatry and Psychology Mayo Clinic Rochester, MN United States; 9 Center for Learning and Mastery Bærum Hospital Vestre Viken Hospital Trust Bærum Norway; 10 National Advisory Unit on Rehabilitation in Rheumatology Department of Rheumatology Diakonhjemmet Hospital Oslo Norway; 11 Faculty of Health VID Specialized University Oslo Norway; 12 Regional Advisory Unit on Pain Oslo University Hospital Oslo Norway

**Keywords:** Web-based interventions, eHealth, mobile apps, evidence-based, user-centered design approach, service design, chronic pain, cognitive behavioral therapy, acceptance and commitment therapy

## Abstract

**Background:**

Chronic pain conditions are complicated and challenging to live with. Electronic health (eHealth) interventions show promise in helping people cope with chronic illness, including pain. The success of these interventions depends not only on the technology and intervention content but also on the users’ acceptance and adherence. Involving all stakeholders (eg, patients, spouses, health care providers, designers, software developers, and researchers) and exploring their input and preferences in the design and development process is an important step toward developing meaningful interventions and possibly strengthening treatment outcomes.

**Objective:**

The aim of this study was to design and develop a user-centered, evidence-based eHealth self-management intervention for people with chronic pain.

**Methods:**

The study employed a multidisciplinary and user-centered design approach. Overall, 20 stakeholders from the project team (ie, 7 researchers, 5 editors, 7 software developers, and 1 user representative), together with 33 external stakeholders (ie, 12 health care providers, 1 health care manger, 1 eHealth research psychologist, and 17 patients with chronic pain and 2 of their spouses) participated in a user-centered development process that included workshops, intervention content development, and usability testing. Intervention content was developed and finalized based on existing evidence, stakeholder input, and user testing. Stakeholder input was examined through qualitative analyses with rapid and in-depth analysis approaches.

**Results:**

Analyses from stakeholder input identified themes including a need for reliable, trustworthy, and evidence-based content, personalization, options for feedback, behavioral tracking, and self-assessment/registration as factors to include in the intervention. Evidence-based intervention content development resulted in one face-to-face introduction session and 9 app-based educational and exercise-based modules. Usability testing provided further insight into how to optimize the design of the intervention to the user group, identifying accessibility and a simple design to be essential.

**Conclusions:**

The design and development process of eHealth interventions should strive to combine well-known evidence-based concepts with stakeholder input. This study, designing and developing the pain management intervention EPIO, illustrates how a stakeholder-centered design approach can provide essential input in the development of an eHealth self-management intervention for people with chronic pain.

**Trial Registration:**

ClinicalTrials.gov NCT03705104; https://clinicaltrials.gov/ct2/show/NCT03705104

## Introduction

### Background

Chronic pain conditions are often multifaceted and difficult to manage, involving physiological and psychological and social challenges for those affected [1-4]. Given this complexity, chronic pain can also be perceived as unavoidable, unmanageable, and challenging to disengage from [5].

Psychoeducational individual or group-based interventions with cognitive behavioral (ie, cognitive behavioral therapy; CBT) [6] and/or acceptance and commitment (ie, acceptance and commitment therapy; ACT [7]) approaches supporting self-management have been shown to be effective. CBT entails an integrative approach combining cognitive and behavioral change techniques, focusing on challenging and changing unhelpful thoughts and behaviors with a goal-oriented, problem-solving approach and enhancement of coping strategies [8]. ACT was initially proposed as a new generation of CBT, focusing on the role of acceptance and mindfulness rather than cognitive change; aiming to increase psychological flexibility; and centering around acceptance, awareness of the moment, and a commitment to values and direction [9]. Both types of interventions can improve a person’s quality of life, pain acceptance, functioning, and self-efficacy while also having the potential to reduce pain, depressive symptoms, and distress [10-18]. Unfortunately, such in-person interventions are not always offered or available [5], and multiple barriers to attendance may be present for people living with chronic pain conditions.

Electronic health (eHealth) interventions, referring to health-related interventions distributed through technology, have shown promising results in helping people cope with health-related issues and chronic illness, including pain [19-25]. eHealth interventions have the potential to offer patients easier access to illness management when convenient and most needed and at the patients’ own pace [26,27]. Given the potential for flexibility in use, eHealth interventions may introduce more cost-effective treatment options, supplementing usual care and even reducing the need for direct involvement from health care providers [28,29]. This could be particularly important when dealing with chronic illness, such as pain, as individuals living with chronic illness usually have the need for, and responsibility of, day-to-day management of their own illness [13]. Several studies have pointed to great potential for the use of eHealth in chronic pain management [21,23,24,27,30,31]. However, a number of challenges have been associated with such interventions. One significant challenge is the lack of guidance and involvement of health care providers and intended users in the development process [21,27,32-37]. This has resulted in a gap between the commercial and scientific sides of eHealth, with products often being developed in response to technological innovations rather than evidence-based knowledge and/or user needs [27,33,38]. There is a need for more attention on how to develop and translate or transform existing face-to-face interventions into electronic formats at the same time focusing on the actual needs of patients with pain [27]. Researchers have made recommendations for utilization of a more user-centered design approach, ideally involving all stakeholders (eg, patients, health care providers and caregivers, pain and eHealth researchers, and designers and information technology [IT] developers) in the eHealth intervention development process from the early idea initiation to the final intervention evaluation [5,27,39]. Despite these recommendations, end users and other stakeholders are still rarely involved in the early development process of eHealth interventions [32,39]. This could potentially be at least partially because of a lack of information and frameworks on *how* to involve stakeholders in the development process [39].

Additional challenges include low adherence and high attrition rates in eHealth interventions [39-43]. For eHealth interventions and development processes to be successful, a focus on the *entire* person (ie, a holistic view), including relationships, context, and intervention setting, is necessary [39,44,45]. This includes identifying facilitators and barriers for use and exploring implementation issues from an early stage on [46-49]. Addressing these issues will likely contribute to development of more user friendly, meaningful interventions for patients and can potentially improve treatment outcomes for patients living with chronic pain [27,39,44,46].

This study is part of a larger project aiming to design, develop, and test the effectiveness of a user-centered, evidence-based eHealth self-management intervention for adults with chronic pain (clinical trial registration: NCT03705104). In the first step of the larger project, users’ everyday challenges and attitudes toward eHealth technology, as well as their needs and requirements for a potential eHealth pain management intervention, were explored through individual interviews with people with chronic pain and their spouses [50]. Individual interviews with health care providers have also been conducted focusing on the same issues (to be published elsewhere). Patients and spouses in the initial study reported a broad spectrum of everyday challenges, including physical, psychological, and social challenges, such as fatigue, grief, guilt, and social- and work isolation, and participants anticipated that eHealth technology would be a positive and accessible option for pain management support [50]. The study found that patients’ needs in relation to an eHealth pain management intervention can be summarized in 3 main areas: (1) need for reliable knowledge about pain and pain management, including access to useful coping skills and exercises; (2) support in finding balance in everyday life, physically and mentally, through increased awareness and simple documentation (ie, ability to track variables such as sleep, mood, physical activity, and pain); and (3) social support, including peer support forums and advice on how to communicate with others, such as family, friends, and health care providers [50].

### Objectives

Building on the recent findings [50], this study aimed to design and develop a user-centered, evidence-based eHealth self-management intervention for people with chronic pain. This paper includes descriptions and results from the development process, including results from workshops with all involved stakeholders (eg, patients, spouses, health care providers, researchers, software developers, and user representatives), intervention content development and usability testing. The ultimate goal was to develop an evidence-based intervention that was acceptable to users (ie, well received, suitable, user friendly, attractive, and meeting needs) [51] and had the potential to produce changes in quality of life for people with chronic pain.

## Methods

### Study Design

The design and development process entailed a multidisciplinary and user-centered design approach [39,52,53]. The project utilized well-established cognitive behavioral pain management concepts shown to be effective for people with chronic pain [11,14,16,54,55] and incorporated concepts of a participatory design approach [56] to ensure that the intervention would be acceptable (ie, well received, suitable, user friendly, and attractive) and designed in line with patients’ needs and context of use.

The intervention development was led by the study principal investigator (PI; LSN), who is a clinical psychologist with health psychology specialization and long-standing experience within chronic pain and cognitive behavioral treatment approaches for medical patients. The multidisciplinary project team consisted of health care researchers (ie, PhD-level psychologists and registered nurses), an editorial group ensuring that content and material was presented in an understandable way, a software team (ie, software developers and a designer), and a user representative (ie, person with chronic pain experience). The team met weekly (sometimes more often) during the development process. See Table 1 for an overview of the project team members with their project-related expertise.

**Table 1:** Overview of multidisciplinary project team (N=20).

Grouping	Total number, n	Pain expertise, n	Electronic health expertise, n	Licensed health care providers, n
Health care researchers	7	6	5	5
Editorial group	5	0	5	2
Software team	7	0	7	0
User representative	1	1	0	0

Patients and other stakeholders, including spouses of patients with chronic pain, and collaborative partners (ie, interdisciplinary health care providers, health care managers, and researchers working at collaborating institutions such as hospitals, municipality health care services, and universities), all with long-standing experience on chronic pain issues, were also involved in the development process. Service design methods, utilizing a user-centered, sequenced, cocreative, and holistic development approach [57] were used to facilitate co-design and high user engagement throughout the development process.

The pain management intervention was developed in an iterative process involving systematic evaluation throughout as suggested by the Center for eHealth Research and Disease Management comprehensive roadmap approach for the uptake and impact of eHealth technologies [39].

**Figure 1:**
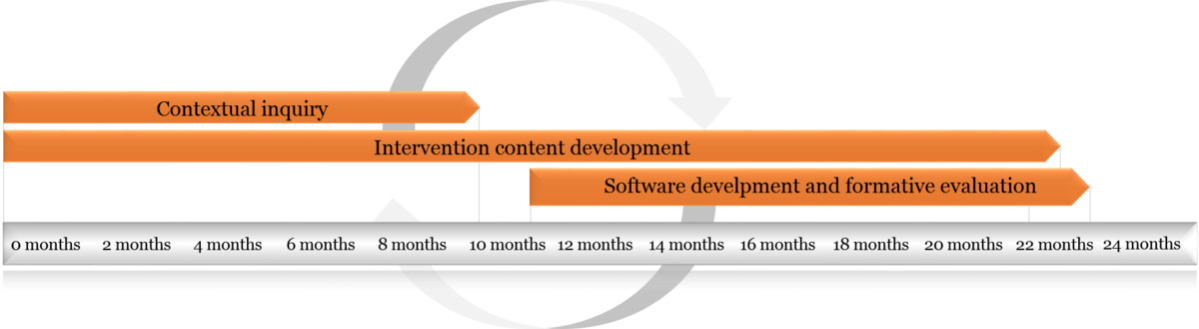
Study overview and development timeline.

The intervention was developed in iterative processes, as shown in Figure 1, through a combination of (1) contextual inquiry and co-design processes collecting input from people with chronic pain, their spouses, health care providers, and eHealth experts; (2) intervention content development, where content was developed by members of the project team based on evidence-based CBT and ACT concepts for chronic pain self-management; and (3) iterative software development and formative evaluation, including low- and high-fidelity prototypes development and usability testing.

#### Recruitment

To be eligible for study participation, the *patients* had to be 18 years or older, having experienced chronic pain for 3 months or more, and had to be able to communicate in Norwegian. Patients were encouraged to participate regardless of age (≥18 years) and gender. *Spouses* had to be married or cohabitating with one of the participating patients.

Patients and spouses were recruited through study information published on the Web as well as through national project collaborators and local and national pain associations. In addition, patients and spouses participating in the initial interviews investigating patients’ needs and requirements for eHealth interventions [50] were invited to participate in this study, as they already had experience with the topic and could potentially add another layer to the design discussions.

#### Data Collection and Analysis

Data were collected from pain management courses, workshops, and usability testing. Then, to ensure that the collected material provided essential input into the ongoing development process, collected data were first analyzed by means of rapid analysis [58]. This included summarizing data from voice recordings and recorded notes before sharing and discussing the material in the project group (including the development team). Following this process, to ensure a thorough scientific development and that no themes were overlooked, a more in-depth analysis of the material took place using directed content analysis [59]. In this process, the material was coded into predefined categories, containing development suggestions and requirements from the participants, including input for content, design, and functionality.

### Intervention Development

#### Contextual Inquiry: Data Collection

A contextual inquiry [39] initiated the development process to gather information about the intended users, their needs and requirements for acceptability, and the environment in which the intervention was intended to fit, building on previously gathered information [50].

#### Pain Management Course Observations

To gain an insight into health care services offered in the study area, as well as to gain additional information related to patients’ needs and experiences, the first (ILS) and second (CV) authors observed 5 different pain management courses available in local patient education centers and hospitals. Notes and summaries were recorded during and after the course observations. The information gathered was summarized into 3 categories: (1) topics covered in the courses, (2) information shared by course participants (ie patients), and (3) themes that potentially could be incorporated into the app. This information was shared with the project team to increase knowledge about the patient group and the pain management courses with the rest of the project team members.

#### Developing Personas and Patient Journey Maps

**Figure 2:**
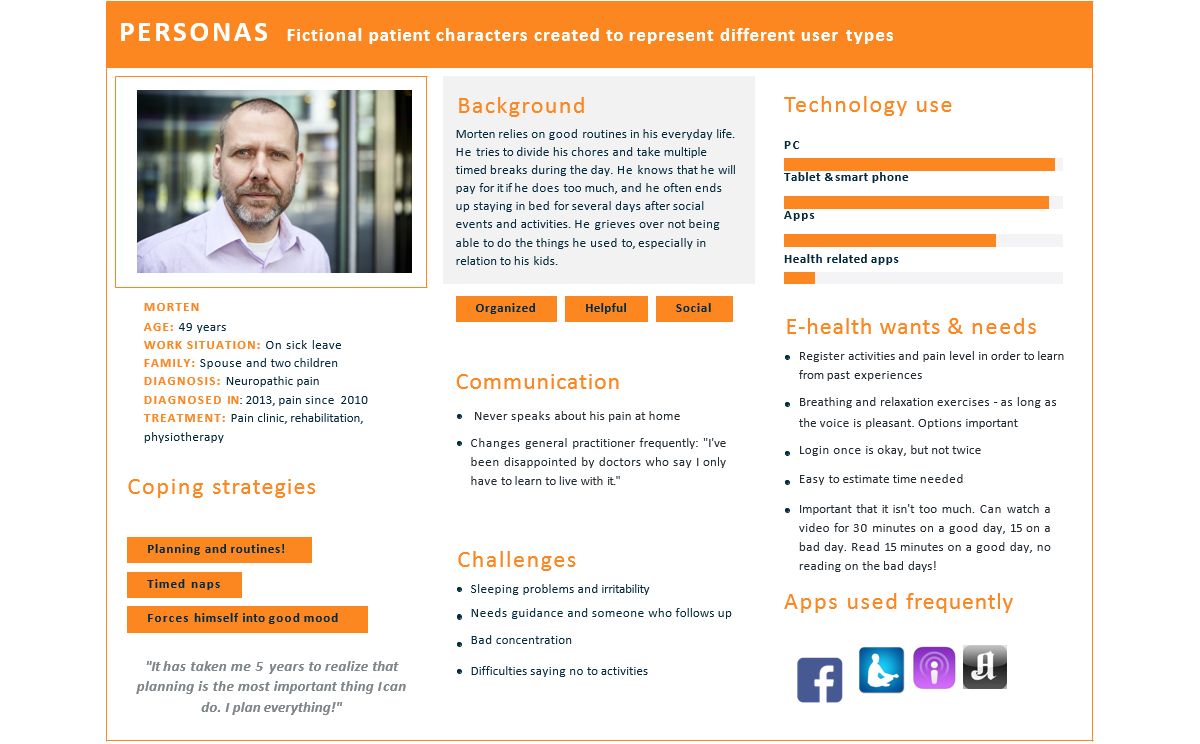
Illustration of a study persona.

**Figure 3:**
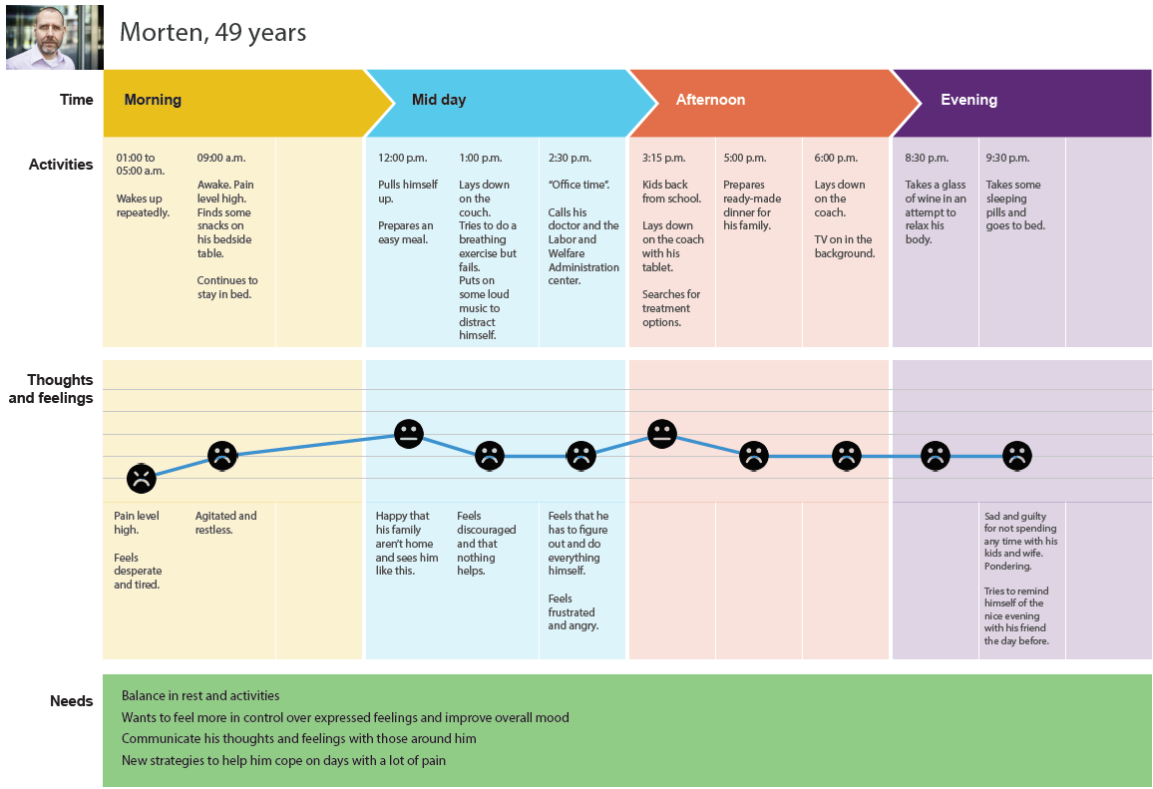
Illustration of a patient journey map during a typical day with pain.

On the basis of existing research recommendations [39,60], service design methods [57] were used to facilitate user engagement. Five personas (ie, fictional but representative patient profiles; see example in Figure 2) and 2 *patient journey maps* (ie, *roadmaps* inspired by customer journey maps [57]; see Figure 3) visualizing typical days in the patients’ lives were developed based on previous findings [50] for the design and development process. Personas included background information (ie, stories to give each persona more depth), coping skills and everyday challenges, an overview of technology skills, and the persona’s needs and requirements in relation to the eHealth intervention. The journey maps described a *typical day* in a patient’s life. Personas and journey maps informed the development process and project team members about *typical* end users and their daily challenges, needs, and requirements to bring the *patients’ voices* to the forefront in the development process. Personas and journey maps were also used in the upcoming workshops as illustrative scenarios that the participants could use when discussing possible design and functionality options. For illustrations, see Figures 2 and 3.

#### Workshops

Contributing to the contextual inquiry, stakeholders were invited to discuss their needs, requirements for acceptability, ideas, and possible challenges related to the eHealth project. This was done successively through (1) 5 workshops with participants from collaborating institutions and the project team and (2) 3 stakeholder workshops involving all stakeholders (ie, people with chronic pain, spouses, health care providers, researchers, editors, and the software team). Service design methods, including the use of scenario tasks, personas, and journey maps [57], were used to facilitate participant engagement. The workshops are described in the following sections.

#### Workshops With Collaborating Partners

First, health care providers, a health care manager, and an eHealth research psychologist from collaborating institutions (ie, hospitals, universities, and primary health care services) were invited to participate in workshops together with the project team.

In the first workshop, participants were separated into smaller groups consisting of health care providers with a variety of professional backgrounds (eg, registered nurses, psychologists, and social workers) and individuals from the project team (including eHealth researchers and software developers). Group discussions were timed and led by group facilitators from the project team. Notes from the group discussions were recorded and materials such as post-it notes and drawings from group tasks were collected. The material was categorized by the first author (ILS) and another project team member using a rapid analysis approach [58] to ensure rapid and continuous input on the development process, broadly sorting the material into idea clusters based on content and similarities.

Information gathered in the first workshop provided guidance for the subsequent 4 workshops, which focused more specifically on the development process (ie, content and software development), with workshop 2 focusing on adherence and design elements and workshops 3 to 5 focusing on content development and how to best present the psychoeducational content. Notes were taken and analyzed using a rapid analysis approach [58].

#### Workshops With All Stakeholders

Building on workshops with collaborative partners, all stakeholders (ie, people with chronic pain, spouses, health care providers, researchers, editors, and the software team) participated in 1 out of 3 stakeholder workshops. The main purpose of these workshops was to elicit ideas on design and content features and to further explore users’ requirements for an eHealth pain management intervention, with each workshop informing the next. The first stakeholder workshop was arranged with only patients participating, together with members from the project team (ie, the designer and 2 researchers, including the first author). In stakeholder workshops 2 and 3, spouses and health care providers from collaborating institutions were also included in addition to editors and software team members. Participants were divided into multidisciplinary groups of 5 to 6. A brief presentation of the personas and journey maps developed initiated each workshop to provide all stakeholders with an overview of findings in the development process so far and to provide a collective understanding of the target patient group. The personas were also updated during the workshops based on participant feedback.

In the first workshop, more time was spent on discussing the personas, whereas stakeholder workshops 2 and 3 focused more on design and functionality aspects. As a starting exercise, participants were asked to reflect upon what makes an app *good or bad* and to discuss usability and acceptability aspects within their groups. Potential design features were then briefly presented to support stakeholders when participating in the design discussions. The participants were then asked to reflect and discuss which design features and elements were most important to them in a priority task, where participants had to choose between different design elements and features. The final part of the workshops included a collective design task, using scenarios, the personas, and patient journey maps to discuss possibilities related to an eHealth self-management intervention (ie, content, functionality, design, and usage) before finally looking at potential barriers for use.

All stakeholder workshops were facilitated by the first author in collaboration with other project team members (KH, HS, MW, JM, and YI), and each group discussion was audio taped. The material was first summarized by the first author using rapid analysis [58] and focusing on ensuring that the material provided essential input into the ongoing development process. The material was later transcribed verbatim to conduct a more thorough analysis using directed content analysis [59] to ensure a thorough scientific process in material identifications. The material was first sorted into broad categories representing requirements for (1) content, (2) functionality, (3) design, and (4) barriers for use. Data were extracted and compared across the different workshops, looking for similarities and differences in the material.

#### Intervention Content Development

A vital goal in this study was to identify evidence-based topics and aspects from recognized cognitive behavioral and acceptance and commitment pain management strategies; then, develop the intervention content and integrate findings and content with a user-centered approach, and subsequently, modify findings to create a new technology-based pain management intervention for people living with chronic pain.

Intervention content development was led by the project PI (LSN), in close collaboration with the other experienced pain management project team members (ie, co-authors KS, LW, EM, KW, HE, and OK), assisted by 3 editors (MW, EB, and HS) and the project-specific user representative. Following the initial workshop with collaborating partners, the intervention content development group first examined the existing literature in the clinical and research area, then discussed the findings and compared notes also based on clinical pain management experience within the group. Intervention content material was then developed based on evidence-based topics and aspects from recognized CBT and ACT pain management [61-64], tested and user tested, and then adjusted and adapted accordingly in continuous iterations. Throughout the process, intervention content was shaped for an electronic format to support usability and ease of use for the end users. The intervention content development underwent numerous iterations (ie, number of iterations varied depending on topic/module) to certify that it used appropriate, therapeutic language; was presented in brief and easily understandable sentences; and was suitable for small screens.

#### Software Development and Formative Evaluation

On the basis of content development and stakeholder input, a low-fidelity paper prototype of the software was developed. The prototype was tested within the development team with involvement from eHealth experts and the project team, then adjusted and implemented electronically to simulate the app idea. To strengthen acceptability, the simulated prototype was subsequently tested by the project team user representative, hospital-employed healthy volunteers, and 1 external patient before full-scale usability testing.

#### Technical Architecture

EPIO is distributed as a native app for iOS and Android through the official app stores, and it is implemented using Web technology in a Cordova container. All information stored locally is encrypted with the Advanced Encryption Standard (AES) algorithm in Galois/counter mode before it is written to a local SQLite (a relational database management system) instance. The key used for encryption is 256 bits long, and it is generated the first time the app runs. Between invocations of the app, it is wrapped using the AES- key-wrapped algorithm with a wrapping key derived from the user’s personal identification number (PIN) and stored on the device’s keychain. As the keychain itself requires the device to be protected with a PIN, the role of the app’s own PIN is to enable the user to secure the app even when using it on a shared device. Usage logs (navigation, use, and use of functionality) and self-assessments are sent over an encrypted channel to a secure server for later analysis by the research project staff.

Technical decisions were executed only after discussions in the project team (ie, researchers, health care personnel, eHealth experts, software design, and developers and user representatives).

#### Usability Testing

Building on feedback and discussions within the project team, high-fidelity prototypes were developed, including a start page, menu page, and intervention modules. A diverse group of users (ie, variety in age/gender), including hospital-employed healthy volunteers and people with chronic pain, participated in the testing.

The high-fidelity prototype usability tests were videotaped and conducted face to face by a facilitator (ie, editor/eHealth expert) and an observant (ie, either the first author, the designer, or another project team member). A think aloud methodology [57] was used to actively engage the participants and elicit continuous feedback, with participants describing their actions and immediate thoughts for each step. The observer took notes throughout the testing. Summaries of observations were completed and transferred into a table by the facilitator and observer following each testing, containing information related to (1) usability issues, (2) possible solutions, (3) who reported this issue (ie, number of users), and (4) other input. This provided a rapid and continuous yet structured feedback into the development process. As a supplement toward establishing acceptability, participants completed Sauro’s System Usability Scale (SUS) [65]. This was done at the end of the usability testing after the facilitator and the observer had left the room. The SUS measures usability and satisfaction on a scale from 1 (strongly disagree) to 5 (strongly agree).

The summaries from the usability testing were discussed within the project team and new sketches and decisions for the next development phase were conducted, with the project team discussing and prioritizing changes. The collected material was later examined more in depth through content analysis [59] to potentially identify themes overlooked in the initial rapid analysis [58]. In this process, the material was sorted into broad categories looking at (1) usability and flow, (2) functionality and customization, (3) intervention content, and (4) design and language.

#### Security and Privacy Considerations

The intervention program was developed at a major medical center in Northern Europe. The design and development were in accordance with the European General Data Protection Regulations of 2018. The study, including a risk assessment analysis of the app, was approved by the institution’s Department of Information Safety and the institutional review board (approval number: 2017/6697). Informed consent will be obtained from all users of the app-based program.

## Results

### Participants

**Figure 4:**
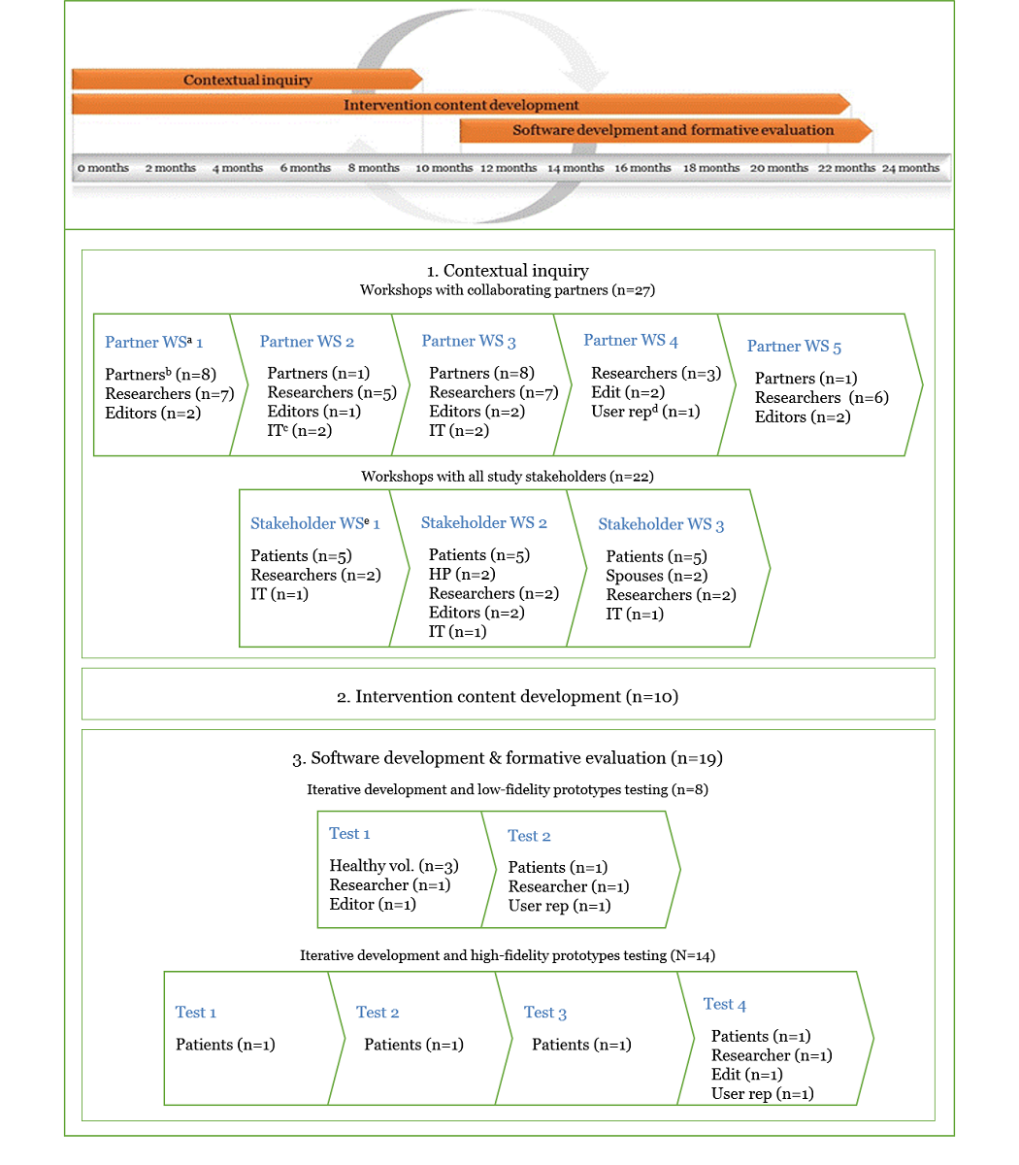
Overview of study timeline, intervention development process, activities, and participation. Partner WS: collaborating partners; Partners: collaborating partners; IT: person(s) from software team (ie, developers and designer); User rep: user representative; Stakeholder WS: stakeholder workshop. The test facilitator and observers are not counted as participants and included in the n for software development and formative evaluation.

A total of 33 participants participated in the study design and development process (ie, workshops and content and software development), including 12 health care providers, 1 health care manager, and 1 eHealth research psychologist from collaborating institutions, as well as 17 patients and 2 of their spouses together with the project team (see Table 1 in the Methods section). For details, please see Table 2 for collaborating partners’ background and expertise, Table 3 for patient demographics, and Figure 4 for a complete overview of the intervention development process, including activities and participation.

All participants, 3 men and 11 women, from collaborating institutions had extensive experience working with people living with chronic pain. They represented a variety of professional backgrounds, with the majority working as licensed psychologists within chronic pain management. In addition, 9 had experience in research and 3 had eHealth expertise.

The majority of the patients had experience with a variety of treatments, ranging from primary care and physical therapy to more specialized treatments and rehabilitation in secondary and tertiary care settings. All participating patients owned a smartphone and had access to a computer and/or tablet. Most of the patients used apps daily, which were either installed by themselves or someone in their family, though several were not familiar with the concept of apps and did not know the difference between a webpage and an app. Many of the patients participated first in 1 out of the 3 workshops and then also later during software development and formative evaluation. The 2 male spouses had been part of the initial interviews [50].

**Table 2:** Overview of background and experience of participants from collaborating institutions (N=14).

Health care background	Total number, N	Pain expertise, n	eHealth expertise, n	Research expertise, n
Nurse	1	1	0	1
Psychologist	7	7	1	5
Physician	1	1	0	1
Social worker	2	2	1	1
Occupational therapist	1	1	0	0
Nonlicensed partner	2^a^	1	1	1

^a^Health care manager and eHealth research psychologist.

**Table 3:** Patient demographics (N=17).

Characteristics		Values, n (%)	
**Gender**			
	Male		2 (12)
	Female		15 (88)
**Age (years), mean age=51 years**			
	20-35		2 (12)
	36-50		5 (29)
	51-59		7 (41)
	60-75		3 (18)
**Type of pain (primary diagnosis reported)**			
	Neck and/or back pain		5 (29)
	Nerve pain/neuropathic pain		5 (29)
	Fibromyalgia		3 (18)
	Migraine		2 (12)
	Others		2 (12)
**Reported years living with pain**			
	0-5		2 (12)
	6-10		6 (35)
	11-17		3 (18)
	18-26		4 (24)
	≥27		2 (12)
**Employment status**			
	Working/studying full time		4 (24)
	Working/studying part time		3 (18)
	On disability benefits		8 (47)
	Retired		1 (6)
	Nonworking		1 (6)

### Pain Management Course Observations

The 5 pain management courses observed covered topics such as pain physiology, coping skills, and psychosocial challenges. They had different structures, the majority being 1- or 2-day courses and 1 occurring weekly over a period of 3 months. The pain management courses covered topics such as pain physiology, coping skills, and psychosocial challenges. The level of information shared by patients during the courses varied, depending on whether the type of course they attended had allocated time for discussions or mainly focused on educational information. However, the patients did describe a general need for more peer support in their everyday lives. Topics otherwise emphasized by participating patients included grief, guilt, anxiety, and negative thoughts as well as issues related to communication with health care providers, spouses/family, and others. Participants generally seemed pleased with the courses they attended, even though several patients emphasized the need for more continuous support and help in everyday life.

### Workshops With Collaborating Partners

Workshop findings showed that participants from collaborating institutions (ie, health care providers and eHealth experts, n=14) were generally positive toward an eHealth intervention for people with chronic pain and saw it as an opportunity to make psychological and educational treatment more accessible to patients during and after treatment. Health care providers pointed to the importance of reliable, evidence-based educational information. Please see Table 4 for content needs and rationales expressed during these workshops.

**Table 4:** Results from workshops with collaborating partners (N=14).

Content needs and topics	Rationale
Reliable, trustworthy, evidence-based knowledge	Provide evidence-based, trustworthy information to patients, giving them a better alternative to online forums and other nonscientific channels
Focus on psychological health	Increase patient’s awareness of the association between chronic pain and psychological challenges
Activity pacing	Support patients in implementing activity pacing strategies in everyday life, including through exercises
Self-assessment/registrations	Increase awareness about personal activities and positive/negative health behavior (eg, amount of sleep and physical activity)
Communication	Include advice on how to best communicate personal struggles, potentially adding direct contact with health care professionals as a functionality
Social support	Adding some form of option for social contact with peers

Potential barriers in the development process, as well as for the final product, were identified as (1) time challenges, referring to the amount of time it takes to develop eHealth interventions and whether or not health care providers had time to participate in the development process; (2) defining the most relevant and beneficial intervention content; (3) patient involvement in the development process; and (4) privacy and security issues.

### Workshops With All Study Stakeholders

The 3 stakeholder workshops identified that patients (n=17), spouses (n=2), and health care providers (n=2) supported many of the same thoughts and ideas as health care professionals and other collaborating partners participating in the initial 5 workshops. The patients particularly emphasized the need for an intervention that gave them positive input in their daily lives. They did not want reminders of what they could *not* do, for example, being asked to do *impossible* exercises or being asked to set *unrealistic* personal goals. Feeling guilty, grief, achieving balance in everyday life, getting support, being present and being useful while taking care of oneself were topics mentioned by all participating groups. Content topics suggested and discussed by participants included updates on recent scientific findings; information about health-promoting behavior (ie, sleep, nutrition, and physical activity); and advice on how to prioritize and set limits, support and information on acceptance, and exercises promoting energy and awareness, such as breathing and relaxation exercises.

Regarding the end product/solution and what it should look like, the participants had a broad range of functionality suggestions and demands. All external stakeholders pointed to personalization, that is, adjusting the intervention based on individual needs and personal preferences. For example, customization and simple behavior trackers were pointed out by some as important features for a chronic pain eHealth intervention. At the same time, several patients, spouses, and health care providers emphasized that too many options for choice could potentially be perceived as overwhelming. The use of gamification elements (ie, application of game-playing elements such as avatars, points, and badges) was viewed as a potentially important and motivating option by collaborating partners and the software team. However, the participating patients did not identify gamification as important compared with other potential elements and features of the solution. Too many or too bright colors, cartoons, or sound effects were described by several patients as potentially challenging for them, especially when experiencing a lot of pain. Many stated that the use of such elements is for *younger people and kids*, and some of the patients also described having stopped using certain apps *because* of such elements.

Health care providers from collaborating institutions focused more on sharing functionality (ie, possibility of sharing health information with health care providers) than did patients. Despite seeing sharing possibilities as something positive, patients were skeptical as to how this could work and found the option unrealistic given the limited time available for health care providers, and their impressions that health care providers often work nonstop with no availability to respond to email/phone calls during a full workday. When asked what mattered most to them, the patients preferred an intervention that could give them personalized suggestions for exercises and content related to the issues and areas they described as challenging. Many patients also emphasized this as one of the main reasons for wanting simple ways to self-assess or track behaviors (eg, for sleep and activity), wanting the intervention to suggest exercises based on their own personal behavioral patterns.

Each stakeholder workshop also involved a priority task (ie, choosing between different design elements and features), where participants voted (each with a maximum 3 votes in addition to 2 group votes) on potential design elements and features. See Table 5 for details on the distribution of votes from patients (n=15), spouses (n=2), and health care providers (n=2); whether the design element/feature was included in the final app; why/why not; and a few illustrating quotes.

**Table 5:** Design elements/features: priority task voting, elements/features included, details, and illustrating quotes.

Design element/feature	Description	Votes, n	Included in the final app (yes/no) and details/justification	Illustrating quotes
Customization and personalization	Customize how things are presented/look in the app. For example, you can customize colors, styles, or specific parts of the app that you want to use.	14	Yes. The features My page and My favorites were included to allow for personalization and easy access to preferred content. In addition, the sequence of some of the modules could be individually chosen, to allow for more individual preference.	“For me, it is very important that it is individually tailored/customized.”
Behavioral trackers	Map/log what you do to see connections and opportunities for change.	13	Yes. Daily self-assessment/registrations of pain, sleep, rest, activity, and mood were included as optional features for those preferring to track all/some of these factors.	“Today, I've been in a lot of pain, but I don’t know why [...] The registrations I’m looking for will tell me why I have so much pain every Thursday.”
Feedback	Get feedback from the app. For instance, by telling you what you have achieved lately or show you new ways to do things.	10	Yes. Several of the exercises in the app allow for registration of current habits/activities and give suggestions for new ways to do things.	“I think it should, in a way, replace a personal coach [...] and be able to provide feedback, and discuss with me. What went well, what went wrong.”
Automatic tailoring	The app automatically adapts to your personal use. For example, you can bring up content and exercises according to your previous preferences.	10	Yes. The app gives the users suggestions for modules and exercises to try based on their marked favorites.	“You may receive quicker feedback if it is automated, as health care personnel go home at 4 pm.”
Visualization	Visualization is used to present content in an engaging and visual way. This can be through the use of animations, cartoons, graphs, etc.	8	Yes. Illustrations and photographs are used in the app to support the content but are presented in a *muted* way so as not to appear overwhelming or challenging. Graphs, illustrating the users’ behavior tracking, were also implemented.	“I imagine some pictures of famous places that give me energy, people or animals that give me energy, and nature, that gives me energy.”
Communicating with health care professionals	Communicate with health care professionals, for example, by sharing information, asking questions, or receiving feedback.	6	No. Not prioritized because of conflict with the desire for easy access by means of a 4-digit personal identification number, and the desire for an app that can serve as a stand-alone self-management program.	“When you have this kind of an app, it is important that when you push the button, you get right in [without high-level log-in procedures], and especially when you are not feeling good.”
Communicate with peers	Communicate with peers/other users, for instance, via forums or share achievements with other users of the app.	2	No. Not prioritized because of potential negative impact, conflict with the desire for easy access, and the notion that this would require a larger user base than planned study inclusion.	“Social contact with other users, I think it can be very negative. You can so easily pull each other down.”
Avatar	Create your own avatar, that is, a person you can be/that follows you in the app. You can customize it to look the way you want, for example, by looking like your favorite animal.	0	Yes. On the basis of eHealth expert input and existing research [41,42], the *buddy* EPIOS (a bird) was included as an engaging element to stimulate engagement and adherence.	“It made me think of children when I saw it.”
Using metaphors	Metaphors can be used as a motivational way of getting through the program/app. For example, let the app be a garden where you can walk around or groom or plant things.	0	No. As the use of metaphors received no votes and was also considered to be a complicating element for the users, this element/feature was not included.	“I did not vote for it” [metaphors] [because I had only three votes to spend and this feature was not important enough for me].
Rewards and trophies	Points and trophies are collected through using the app. For example, you can go up a level when you have collected enough points or get a trophy for strikes, for example, when you have used the app every day for a week.	0	Yes. On the basis of eHealth expert input and findings from existing research [41,42], rewards and trophies were included as engaging elements to stimulate engagement and adherence.	“I’m not very competitive so it doesn’t suit me very well, but I can see that it may be a good thing for others.”

### Intervention Content Development

Existing interview material [50], workshop discussions and usability testing (presented below) indicated that participants preferred a neutral name for the intervention program, encouraging limited use of negative words, such as *pain*, or *too positive* words, such as *positive focus*. During content development, a project team brainstorming and informal *name* *competition* resulted in the intervention being named EPIO, derived from the Greek mythology goddess Epione, the goddess for the soothing of pain.

As evidence-based psychosocial/educational interventions are mainly conducted in person, the decision was made for the EPIO program to contain a face-to-face introduction session, where participants would receive an introduction to the EPIO intervention program, as well as help in downloading the app onto their smartphone or tablet.

Given the significant evidence of potential for support from CBT and ACT for people with chronic pain[10-18], combined with input from all stakeholders, the EPIO intervention was primarily based on CBT (ie, thought and behavior challenging, cognitive restructuring, behavior change, problem solving, and coping) [6,8] but with aspects of ACT (ie, value-based direction, acceptance of pain, and awareness of the present) [7,9], both resting on aspects found essential for pain management [10,18]. The final app-based EPIO intervention program contained 9 modules, as illustrated in Table 6. Each module in EPIO contained educational topics (eg, about pain, balance and activity pacing, thoughts and feelings, health behaviors, and coping during difficult times), as well as brief topic-related tasks and a variety of relaxation-focused exercises (eg, diaphragmatic breathing, progressive muscle relaxation, visualization, mindfulness, and meditation) anchored in existing treatment manuals and findings for chronic pain management [61,64]. The first 5 modules were presented consecutively in a fixed sequence, as the content in each of these modules was considered essential for the subsequent topics. To allow for more individual preference, the sequence of modules 6 to 8 could then be individually chosen, if preferring to do so.

**Table 6:** Overview of EPIO modules and content.

Module #	Module title	Content
0	Introductory session	60 min in-person/group session. Introduction and intervention overview, practical exercise example, and help in downloading and using the intervention.
1	About pain	Introduction to the intervention program, including information about pain and pain management. Coping strategies, fight-or-flight response, and introduction to breathing and relaxation; rationale and exercises.
2	Balance	Activity pacing and planning, introduction to mindfulness, self-care, pleasant activities, EPIO as your toolbox, and progressive muscle relaxation.
3	Thoughts and feelings	Pain, the relationship between thoughts and feelings, recognizing negative thoughts and cognitive distortions, gratitude, and positive thinking. Exercises including challenging negative thoughts, mindfulness, and autogenic muscle relaxation.
4	Stress and coping	About stress, coping, and rationale for stress management and relaxation strategies. Acceptance, active and passive coping approaches, and visualization.
5	What is important to me?	Defining and exploring individual values and goals. Personal role models, self-image, and intruding thoughts. Planning and goal setting. Introduction to meditation.
6	Health behaviors and lifestyle	Health behaviors and health behavior change. Awareness of important health behaviors, including sleep, physical activity, nutrition, and substance use/abuse; rationale and exercises. Stretch-based relaxation methods/exercises.
7	Communication, relations, and social support	Communication, assertiveness, support systems, and social networks. Exercises related to awareness about social support systems, how to strengthen social support, and progressive muscle relaxation.
8	Coping during difficult times	Self-regulation and implementation of coping strategies in everyday life. Pain, frustration and anger management, daily use of coping strategies in everyday life. Introduction and use of distraction, visualization, and stretch-based relaxation.
9	Summary and the road ahead	Review and summary, where to go from here and advice for the road ahead.

#### Software Development and Formative Evaluation

**Figure 5:**
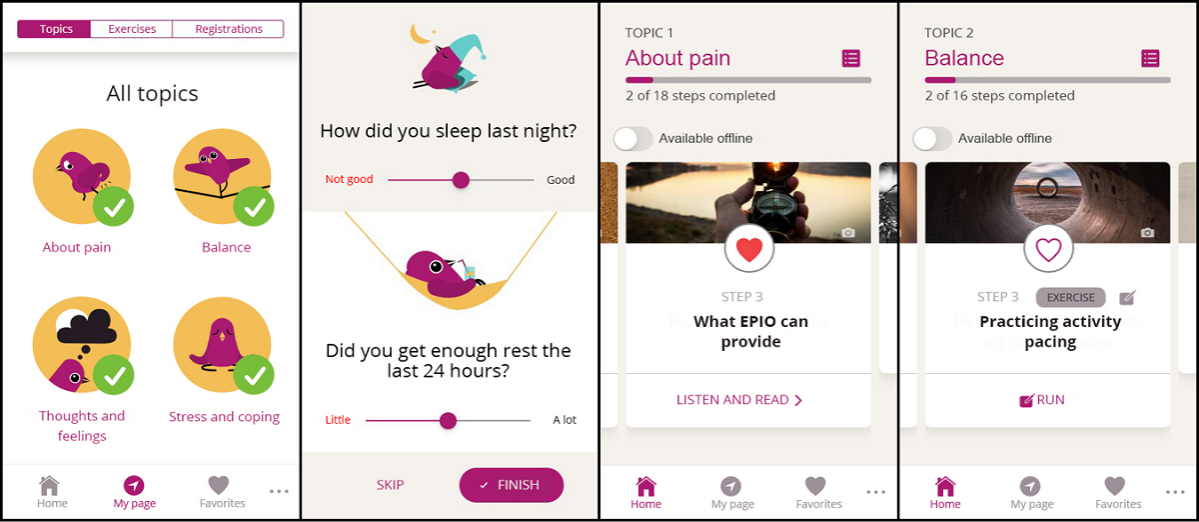
Example screenshots from the EPIO intervention. From left: (1) start page, (2) selective registrations, (3) module about pain, and (4) exercise example.

Stakeholders’ input and 5 iterative rounds of usability testing contributed to adjustments to detect and ensure (1) easy and intuitive navigation, including adding short cuts, introductions, and symbols; (2) language and content issues, including adding more steps to reduce the length of each section, replacing or removing difficult words and terms; (3) implementation of engaging design elements to stimulate adherence, including adding rewards/trophies, as well as an avatar, the *buddy* EPIOS, an animated bird accompanying the users throughout EPIO, and finally d) possibilities for personal preferences and choice, including adding possibilities to choose between reading or listening, and choosing which, if any, variables to track (eg, pain, sleep, activity, mood, and rest). See Figure 5 for screenshot examples.

The usability testing revealed an overall average SUS score of 81.25, indicating a grade A, which equals to excellent system usability [65]. Users reported that the intervention was easy to use, without need for assistance from anyone. Most users reported that they thought they would use the intervention frequently (mean 3.8, median 4).

In response to input from study participants, a number of adjustments were made for the final version of EPIO. Some of these are described in the following sections.

#### My Page

As personalization was emphasized as an important feature, a personal page, *My page* was added to the program. This included an overview of the user’s personal progress in the intervention program in addition to access to personal registrations, illustrated in graphs. In addition, based on the input from eHealth experts and previous research [41,42], a *trophies* section was included in *My page* to stimulate personalization and motivation. Ability to gauge the length of each step and exercise in seconds/minutes was also implemented.

#### My Favorites

Patients expressed a need for easy and direct access to exercises and content that they liked; therefore, a *mark as favorite* feature was added to each step in the program. As usability testing revealed usability issues for this feature (ie, difficulties for users to grasp *how* to mark their favorites), the final program version included introductions presented in a step-by-step manner, with the *buddy* bird EPIOS later reminding users of these steps and the option to add the text *add as favorite* at the end of each step. Usability testing revealed that participants liked the bird EPIOS and the brief summaries and reminders provided by EPIOS.

#### Practicing Mode

CBT typically includes homework between sessions to practice and generalize new skills and behaviors. Therefore, practice and repetition were encouraged in EPIO, and following completion of each module, participants could not open a new module for 3 days. This was done to give users time to practice and implement completed modules, knowledge, and exercises into their daily lives. EPIO provided encouragement for practice, either through suggested steps or through choosing own favorites.

### Security and Privacy Considerations

The EPIO intervention program was developed for people with chronic pain. It was developed at and distributed from a major hospital. Protecting patients and patient information is the responsibility of health care providers and the institution (ie, the hospital) and privacy and security were of essence to consider in the design and development process.

One issue concerned the amount of sensitive information and options related to log-in requirements. Participating patients emphasized the importance of a simple log-in procedure. Most of these patients expressed little concern regarding privacy and security protections, stating that it was more important for them to get an accessible tool they could get *direct access to on a bad day*, referring to days with a lot of pain, *without any hassle or things to remember*, such as high-level access procedures and passwords. This was the case in the stakeholder workshops not only in this study but also in previous patient/spouse interviews [50]. At the same time, however, many of the patients wanted to be able to keep personal notes/diaries, and some also wanted to be able to share their information with their health care provider through the app and/or connect with peers using the app. This would introduce further privacy challenges. Adding functionality such as sharing options would increase the privacy level needed and therefore also increase the security requirements. However, privacy and security are essential in these types of settings, and as ease of use and a simple log-in were identified as one of the most important patient requirements, the solution was to incorporate a simple 4-digit PIN, excluding functionality such as sharing possibilities and personal notes. Users were instead encouraged to use a pen and paper and take notes during some of the themes and exercises (ie, “You may find it beneficial to use a pen and paper for this exercise.”).

## Discussion

### Principal Findings

This study describes the design and development process of EPIO, an eHealth pain management intervention for people with chronic pain. The process combining evidence-based and user-centered approaches is a previously recommended but underutilized approach to eHealth intervention development [27,33,34]. To our knowledge, this is the first study combining evidence-based knowledge with stakeholders’ input to inform the development of an eHealth intervention for self-management of chronic pain.

The EPIO intervention program was developed using iterative processes through a combination of (1) *contextual inquiry and co-design processes*, where input from people with chronic pain, spouses, health care providers, and other collaborating partners was gathered; (2) *intervention content development*, where relevant content topics were identified and intervention content was created based on clinical experience and with inspiration from existing evidence-based cognitive behavioral and acceptance and commitment pain management programs [61-64]; and (3) *iterative software development and formative evaluation*, including low- and high-fidelity prototypes and usability testing. External stakeholders (ie, patients, spouses, health care providers, and other partners from collaborating institutions) described a number of challenges associated with current options for pain management care, emphasizing the potential within eHealth technology and more available sources for pain management strategies. Patients described the need for an accessible solution that fits within their existing everyday routines, giving them a *break*; positive input; and reminders in their daily lives. To meet acceptability and usability needs for the target group, the intervention used easily understandable language, with brief and to-the-point sections made accessible on small screens and mobile phones. Stakeholders also pointed to a need for intuitive and effective functionalities that did not demand too much of the patients’ time, giving them options to choose from and automatic suggestions adjusted to their needs.

### Evidence-Based Knowledge and the Importance of User Involvement: Finding the Right Balance

CBT- and ACT-based psychological interventions have been shown to be effective, improving quality of life, depressive symptoms, and pain acceptance for people living with chronic pain [10,11,13,14,16,66,67]. The goal of this study was to design and develop such an intervention to be delivered in a technological format and on a mobile platform. Seeking to achieve persistent change in a person’s health and overall well-being, intervention programs must be based on evidence-based knowledge, and according to the Medical Research Council’s guidance, all complex interventions should be guided by the latest evidence and appropriate theory [68].

Despite these facts, the development of evidence-based eHealth pain management interventions has been limited [33-35], as has the incorporation of user involvement in these processes. Even evidence-based interventions depend on users’ acceptance, adherence, and overall user fit for interventions to be successful [39]. The lack of user involvement (ie, patients and health care providers) and human centeredness in development of eHealth interventions have been criticized repeatedly [5,21,27,32,34,36,39], and this lack of user involvement in the development process can potentially also explain the high attrition rates and low adherence associated with such interventions [32,40-42]. People simply stop using technologies that do not meet their needs, requirements, or daily routines.

In addition to general user requirements (eg, technology being user friendly and flawless), it is essential to incorporate the needs and requirements of the specific user group. For the participants in this study, that meant the eHealth intervention had to accommodate patients’ varying and often high pain levels, their challenges with feeling guilty and *never doing enough*, and their concentration issues. Stakeholders stated that it was important that the intervention did not focus on the negative aspects of living with chronic pain or provide users with too much information or too much choice, flashy graphics such as sound and animation effects, or cumbersome log-in procedures. Patients wanted positive input in their daily lives through a solution that provided them with useful, effective, and personalized advice on pain management, reminding them to take smaller breaks during the day. Participants also suggested functionalities to register daily activities, sleep, and mood level so that patients could become more aware of how these areas affected their daily life. Some of the patients also wanted an option to register their daily pain level. From a CBT standpoint, this could be viewed as useful, as increasing patients’ awareness and ability to take an active part in one’s own life is crucial. However, the literature has shown that too much focus on the pain itself, for instance, through keeping a pain diary, can be negative and could possibly increase pain interference [69-71]. However, studies have also illustrated the positive sides of pain screenings/registrations. As several participants regarded self-assessment/registrations as important, and this was also voted high on the prioritizing task (Table 5), self-assessment/registrations were included in the initial development.

The design and development process in this study did reveal some disagreements between what was considered important by health care providers and other collaborating partners versus what was considered important by some of the patients. Although health care providers emphasized the need for available, evidence-based, and trustworthy information given to patients, seeing eHealth technology as a positive option for providing patients with such knowledge, patients expressed some conflicting views on the topic. Patients generally agreed that information and content should be trustworthy, yet they kept emphasizing during workshops as well as usability testing that they did not want *too much* information, that they *already knew* a lot about pain and the theory behind pain management, and that they first and foremost wanted effective and quick exercises that could help in their daily lives. This could have been because several patients were recruited from patient education centers and courses focusing on chronic pain, and thus, they already had received a lot of information. However, literature has shown that compared with the general population, people living with long-term conditions report more difficulties with understanding health information in addition to having greater difficulties in engaging with health care providers [72]. This, together with some chronic pain patients’ reported concentration issues, illustrates the importance of providing chronic pain patients with easily accessible information.

Interventions, and perhaps particularly eHealth interventions, have the challenges of enhancing motivation for use, adherence to use, and motivation for continued use. How to best present evidence-based content is a question of user involvement, acceptability, usability, and feasibility. To promote user engagement and continued use, the EPIO intervention had to present the material in a way that met the users’ interests and requirements. Participating health care providers and other collaborating partners with eHealth expertise, as well as software developers, suggested adding gamifying design elements such as rewards and avatars, emphasizing the importance of engaging and motivational design elements and pointing to evidence that shows that the use of such elements and persuasive technology positively affects adherence and well-being [41,42]. However, the participating patients found this less important, and they also expressed concern that the use of such effects could be potentially challenging when in pain. None of the patients voted for such elements in the priority task in the stakeholder workshops (see Table 5). Patients instead stated that they wanted the content presented in a simple way.

On the basis of these findings, it was important to find a balance in the use of design elements, with the final EPIO program including some of these types of elements, such as trophies for progress and continued use; and an avatar/buddy, the bird EPIOS; and providing users with content summaries and brief motivational messages. The buddy bird EPIOS, therefore, has an educational role in the intervention program, in line with what users emphasized as important, but at the same time, EPIOS has a motivational and relational role, in line with participating health care providers and eHealth experts as well as recommendations from existing literature [42].

### Strengths, Limitations, and Future Directions

This study presents some limitations that need to be considered. First, the limited number of male and younger patients (mean age 51 years) might limit the representativeness of the study. Given the large percentage of female patients compared with male patients participating in this study, despite encouraging participation of both genders living with chronic pain, the patient sample can be considered a sample of convenience. However, it should be noted that the prevalence of chronic pain is higher among females compared with males [73], and research also shows challenges in recruiting male participants compared with female participants for intervention programs focusing on self-management [74]. In addition, the participating patients represented a wide range of pain diagnoses, and as chronic pain is more prevalent among people older than 50 years [75], it may be argued that the patients participating in this study were in fact representative of the user group. The fact that some of the patients and spouses had also participated in an initial interview study [50] could potentially also be a limitation, as it is possible that other opinions and perspectives would have emerged if more novel users without prior knowledge of the emerging intervention had participated. However, mutual learning and shared understanding are core concepts within participatory design, as this is the only way to ensure mutual respect between stakeholders, enabling everyone to take part in the shared decision-making process [56]. Patients are not eHealth experts and do not necessarily have the language to articulate what they need from an eHealth intervention. Consequently, using the same sample of participants and giving them enough knowledge about design and development processes may have made it easier for the participating patients to take an active part in development discussions. However, this study did also include new and *naïve* patients with chronic pain to add to previously collected qualitative data.

The software development and formative evaluation may also present some limitations. For instance, every part of the intervention steps/modules was not tested. However, the intervention material was based on the same concepts and foundations, written by the same experienced team [76,77], led by the same PI, and using the same therapeutic language and structure. Therefore, it was considered more important to get users’ feedback on functionality and design, including layouts and how the material was presented, than on every written word. Usability testing was conducted at the project team offices, with a facilitator and observer watching the participants within a limited period. This could have impacted the testing, and it is possible that the usability testing of EPIO captured only some of the potential barriers to continued use over time.

A number of strengths are also evident in the current design and development process. As recommended by existing research [5,27,39] and to ensure trustworthiness [78], the study included a broad range of stakeholders (ie, patients, spouses, health care providers, and eHealth experts as well as researchers, editors, software developers, and user representatives) from the project planning stage and throughout the development process.

The development process was guided by existing development recommendations, using a broad range of service design methods and a user-centered design approach to facilitate cocreation, mutual learning, and shared understanding among the stakeholders involved. Intervention acceptability (ie, well received, suitable, user friendly, attractive, and meeting needs) to users was one of the main goals for the design and development of EPIO. Although the intervention program was developed using a participatory design approach to support the likelihood of acceptability, usability, and feasibility, acceptability will need to be further tested and established in a future pilot test study before instigating efficacy studies. Given the challenge of low adherence and high attrition rates in eHealth interventions [41,42], the development of the current intervention sought to incorporate stakeholder-identified aspects supporting adherence. Whether this turns out to be an effective approach facilitating adherence needs to be evaluated in a future pilot test and subsequent randomized controlled trial (RCT). In addition to high user involvement and stakeholder input, the development process was guided by theory and concepts from well-established cognitive behavioral and acceptance and commitment pain management programs, meeting the requests for eHealth pain management interventions that are based on evidence-based knowledge. This enhances the future potential for positive effect findings. The ultimate goal of the EPIO program is to support improvements in quality of life for people with chronic pain. Therefore, in addition to test usability, feasibility, and acceptability, the next step in the research process will be to examine preliminary efficacy findings in a pilot test before eventually examining the intervention in a full-scale RCT. Together with the high focus on privacy and security aspects, acceptability and efficacy are also likely to increase the potential for poststudy implementation.

### Conclusions

This study offers insight into how to take a user-centered approach to the design and development of an evidence-based eHealth pain management intervention for people with chronic pain. Developing evidence-based eHealth interventions while also involving the voices and perspectives of a variety of stakeholders can be challenging, time consuming, and sometimes even an expensive process. However, continuing to develop and test non–evidence-based, non–user-centered interventions is not a great alternative. Instead, mutual learning and shared understanding become crucial. This study involved patients, spouses, health care providers, and other relevant stakeholders in the design and development process of the eHealth interventions, pointing to important steps for developing useful and meaningful interventions for patients. In addition to informing the potential process of developing an eHealth pain management intervention, this study also provides a practical example of how eHealth interventions can be designed and developed to combine evidence-based material with user-centered requirements. To test usability, acceptability, and feasibility, as well as the potential efficacy of the program, further research is needed and a pilot test is currently underway to optimize the EPIO program in preparation for a full-scale RCT.

## Abbreviations

ACT: acceptance and commitment therapy
AES: Advanced Encryption Standard
CBT: cognitive behavioral therapy
eHealth: electronic health
IT: information technology
PI: principal investigator
RCT: randomized controlled trial
SUS: System Usability Scale
